# Impacto de la composición corporal y del estado psicológico en las funciones ejecutivas de personas con obesidad

**DOI:** 10.23938/ASSN.1113

**Published:** 2025-04-25

**Authors:** Mario Tomé-Fernández, Marina Berbegal-Bernabeu, Miriam Sánchez-Sansegundo, José Antonio Hurtado-Sánchez, José Tuells, Ana Zaragoza-Martí

**Affiliations:** 1 Universidad de Alicante Facultad de Ciencias de la Salud Departamento de Psicología de la Salud Alicante España; 2 Universidad de Alicante Facultad de Ciencias de la Salud Departamento de Enfermería Alicante España; 3 Universidad de Alicante Facultad de Ciencias de la Salud Departamento de Enfermería Comunitaria, Medicina Preventiva, Salud Pública e Historia de la Ciencia Alicante España; 4 Instituto de Investigaciones Biomédicas y Sanitarias de Alicante Fundación ISABIAL Alicante España

**Keywords:** Antropometría, Cognición, Neuropsicología, Obesidad, Salud Mental, Anthropometry, Cognition, Neuropsychology, Obesity, Mental Health

## Abstract

**Fundamento:**

El objetivo es examinar la relación entre variables antropométricas y psicológicas con las funciones ejecutivas en personas con obesidad.

**Método:**

Se reclutaron personas adultas con obesidad en Alicante (España). Se realizó una entrevista para recoger los datos sociodemográficos (sexo, edad, nivel de estudios, estado civil y situación laboral), así como una evaluación antropométrica en la que se midió el peso, el índice de masa corporal (IMC), la grasa visceral, la masa grasa (MG) y la masa muscular (MM). La evaluación psicológica se realizó mediante el DASS-21 y la cognitiva de las funciones ejecutivas mediante el M-WCST (flexibilidad cognitiva), WAIS-IV (memoria de trabajo), TMTA (velocidad de procesamiento) y TMTB (control inhibitorio).

**Resultados:**

La muestra estuvo compuesta por 48 personas, 52% mujeres, y media de edad 47,58 años, más frecuentemente con estudios secundarios, casadas y empleadas. Las mujeres tuvieron mayor peso, IMC y MG (% y kg). Los modelos de regresión lineal múltiple mostraron influencias significativas de las variables antropométricas peso, IMC, grasa visceral, MG y MM en el rendimiento de la memoria de trabajo, la velocidad de procesamiento y el control inhibitorio, así como de la ansiedad en la flexibilidad cognitiva y la memoria de trabajo, y de la depresión en el control inhibitorio.

**Conclusiones:**

Las variables antropométricas peso, IMC, grasa visceral, MG y MM influyeron en el rendimiento de la memoria de trabajo, velocidad de procesamiento y control inhibitorio, mientras que la ansiedad influyó en la flexibilidad cognitiva y la memoria de trabajo, y la depresión en el control inhibitorio.

## Abstract (en)

**Background:**

This study aimed to examine the relationship between anthropometric and psychological variables and executive functions in individuals with obesity.

**Method:**

Adults with obesity were recruited in Alicante (Spain). Participants underwent interviews to collect sociodemographic data (sex, age, educational level, marital status, and employment status) and anthropometric assessments measuring weight, body mass index, visceral fat, fat mass, and muscle mass. Psychological evaluations were carried out using the DASS-21. Executive functions were assessed through the M-WCST (cognitive flexibility), WAIS-IV (working memory), TMTA (processing speed), and TMTB (inhibitory control).

**Results:**

The sample comprised 48 participants (52% female) with a mean age of 47.58 years. Most participants had secondary education, were married, and employed. Women exhibited higher values in weight, body mass index, and fat mass (% and kg). Multiple linear regression analyses revealed that anthropometric variables -weight, body mass index, visceral fat, fat mass, and muscle mass- significantly influenced performance on working memory, processing speed, and inhibitory control performance. Additionally, anxiety levels were associated with cognitive flexibility and working memory, while depression levels were linked to inhibitory control.

**Conclusions:**

Anthropometric variables -weight, body mass index, visceral fat, fat mass, and muscle mass- are associated with variations in executive function performance, particularly in working memory, processing speed, and inhibitory control. Psychological states -anxiety and depression- are related to specific aspects of executive functioning.

## INTRODUCCIÓN

Según la Organización Mundial de la Salud^1^, la obesidad es una enfermedad crónica causada por la acumulación excesiva de grasa corporal que representa un importante riesgo para la salud. Los criterios de clasificación están basados en el índice de masa corporal (IMC), los cuales definen la obesidad grado I con un IMC entre 30 y 34,9; obesidad de grado II con un IMC entre 35 y 39,9; y obesidad grado III (obesidad mórbida) con un IMC superior a 40. En las últimas décadas, la literatura sitúa a la obesidad como uno de los problemas más importantes de salud pública debido a su elevada prevalencia y a sus consecuencias en la calidad de vida, discapacidad y aumento del riesgo de mortalidad^2^.

El informe World Obesity Atlas 2023^3^ muestra que en el año 2020 había 347 millones de hombres y 466 millones de mujeres en estado de obesidad en todo el mundo, lo que implica una prevalencia global del 16,3%. En España, la prevalencia en mayores de 18 años se sitúa en un 15,5% en mujeres y un 16% en hombres^4^. Las causas de la obesidad son multifactoriales e incluyen factores conductuales, ambientales, fisiológicos y genéticos^5^. Estudios epidemiológicos han demostrado una clara asociación entre la obesidad y enfermedades crónicas como la diabetes mellitus^6^, trastornos cardiovasculares^7^ y ciertos tipos de cáncer^8^.

Asimismo, la obesidad se ha relacionado con problemas de salud mental y psicopatología^9^, especialmente con trastornos depresivos y trastornos de ansiedad^10^. La literatura muestra que esta relación es bidireccional. Sufrir trastornos psicológicos aumenta el riesgo de desarrollar obesidad, y la condición de obesidad incrementa la probabilidad de sufrir problemas psicopatológicos^11^. Independientemente del sexo, las personas obesas experimentan niveles de estrés y ansiedad más altos que personas con normopeso^12^.

A nivel descriptivo, varios metaanálisis han concluido que las personas con obesidad conforman un perfil psicológico con mayores niveles de depresión^13^^,^^14^ y ansiedad^15^^,^^16^. Además, estudios longitudinales muestran que las personas con sintomatología depresiva tienen un riesgo significativamente mayor de desarrollar obesidad^17^.

La condición de obesidad no sólo tiene consecuencias a nivel físico y psicológico, sino también cognitivo, ya que supone un factor de riesgo para desarrollar demencias en etapas posteriores^18^. En los últimos años se ha descrito una asociación negativa entre medidas antropométricas y el rendimiento cognitivo^19^. Por ejemplo, la obesidad se relaciona con un menor rendimiento en tareas de memoria episódica, aprendizaje y reconocimiento verbal^20^; memoria episódica visual^21^; capacidad psicomotriz^22^; atención selectiva^23^; y función ejecutiva^24^.

Las funciones ejecutivas constituyen un complejo conjunto de procesos cognitivos necesarios para responder de forma adaptativa a situaciones novedosas^25^. Proporcionan la capacidad de organizar, integrar y manipular la información para tomar decisiones y cumplir objetivos^26^. Un reciente metaanálisis de 72 estudios concluyó que un mayor IMC contribuye a la aparición de déficits en capacidades de inhibición, toma de decisiones, planificación, memoria de trabajo y flexibilidad cognitiva^27^. Estos resultados ponen de manifiesto la importancia del peso en el rendimiento cognitivo y sugieren la necesidad de reducir los índices de sobrepeso y obesidad en la población para preservar las funciones cognitivas a lo largo de la vida.

Por tanto, el presente estudio tiene como objetivo examinar la relación entre las variables antropométricas (peso, IMC, grasa visceral, grasa corporal y masa muscular) y el estado psicológico (estrés, ansiedad y depresión), con el rendimiento ejecutivo (flexibilidad cognitiva, memoria de trabajo, velocidad de procesamiento y control inhibitorio) en personas con obesidad.

## MATERIAL Y MÉTODOS

Estudio transversal realizado en personas adultas con obesidad pertenecientes a dos proyectos de investigación basados en la importancia del entorno epigenético de la obesidad grave (grado III/IV) (PI2021-194) y el papel de la dieta en la intervención de pérdida de peso en individuos con obesidad (FS_FOR140), en la Universidad de Alicante (España) entre septiembre de 2023 y febrero de 2024.

### Participantes

Los participantes fueron reclutados en persona en la Unidad Multidisciplinar de Atención Integral al Paciente Obeso (UMAIO) del Hospital General de Alicante y en el Servicio de Endocrinología del Hospital Universitario de San Juan de Alicante. A todas ellas se les ofreció la posibilidad de participar en el estudio si cumplían los criterios de selección, proporcionándoles información sobre las condiciones del estudio, su carácter voluntario y la opción de retirarse del mismo sin ninguna repercusión. Una vez firmado el consentimiento, se les indicó que respondieran a los datos sociodemográficos en formato electrónico, y se les citó en la Universidad de Alicante.

Las evaluaciones se realizaron en la Facultad de Ciencias de la Salud entre el 25 de septiembre de 2023 y el 16 de febrero de 2024. Se realizó un análisis de la composición corporal para determinar las características antropométricas, y se administraron cuestionarios psicológicos y pruebas neuropsicológicas para conocer el estado mental y cognitivo actual.

Los criterios de inclusión fueron ser una persona adulta de entre 18 y 65 años de edad, con un IMC superior a 30 kg/m^2^. Los criterios de exclusión fueron: padecer una enfermedad oncológica o mental, estar embarazada, y consumir fármacos que pudieran afectar al peso (corticosteroides, topiramato, aGLP1 e iSGLT2).

### Variables sociodemográficas

Las variables sociodemográficas se recogieron a través de un cuestionario específico creado en *Google Forms* el cual contenía preguntas relacionadas con las siguientes variables: sexo (mujer, hombre), edad (años), estado civil (persona soltera, casada, divorciada), nivel educativo/de estudios (primarios, secundarios, superiores), situación laboral (persona empleada, desempleada, jubilada).

### Variables antropométricas

Se utilizó un TANITA MC-780MA P (TANITA Corporation, Arlington Heights, IL, EE.UU.) para analizar la composición corporal. Los datos recopilados fueron: peso (kg), estatura (cm), índice de masa corporal (IMC, kg/m^2^), grasa visceral, grasa corporal o masa grasa (MG, porcentaje y kg), masa muscular (MM, porcentaje y kg).

### Variables psicológicas

Para evaluar el estado mental se empleó la Escala de Depresión, Ansiedad y Estrés 21 (DASS-21)^28^, con tres subescalas (Depresión, Ansiedad y Estrés) con alta consistencia interna (α=0,91 para depresión y α=0,88 para ansiedad y estrés). Contiene veintidós ítems con cuatro opciones de respuesta tipo Likert desde no me ha pasado hasta me ha pasado mucho. Se corrige mediante la suma total del cuestionario y para evaluar cada subescala por separado, se deben sumar las puntuaciones de los ítems correspondientes a cada una. A mayor puntuación general, mayor grado sintomatología psicológica y, por tanto, peor salud mental.

### Variables cognitivas

Se administró un protocolo de evaluación neuropsicológica individual de una hora de duración. Se seleccionaron las siguientes pruebas cognitivas para cada una de las funciones ejecutivas evaluadas:

*Flexibilidad cognitiva*: se define como la capacidad de adaptar el pensamiento y la conducta ante cambios en el entorno, reglas o demandas, con el objetivo de alcanzar objetivos específicos^29^. Para la evaluación de este dominio cognitivo se utilizó el *Test modificado de clasificación de tarjetas de Wisconsin (M-WCST)*^30^. La prueba comienza con cuatro tarjetas situadas en frente de la persona (un triángulo rojo, dos estrellas verdes, tres cruces amarillas y cuatro círculos azules), las cuales sirven como modelo/plantilla. El objetivo de la tarea es que la persona participante clasifique las 48 tarjetas restantes en función de los siguientes criterios: color de los objetos, tipo de figura o cantidad de figuras (color, forma o número, respectivamente). La persona evaluadora, tras cada asignación, proporcionará una respuesta *bien* o *mal*, que condicionarán la siguiente clasificación: *bien* significa que la clasificación es correcta y, por tanto, la persona deberá seguir clasificando las tarjetas por el mismo criterio; *mal* significa que la clasificación es errónea y, por tanto, la persona deberá buscar el criterio correcto en el siguiente turno. Cada seis tarjetas clasificadas correctamente se completa una categoría. El tiempo aproximado de administración es de seis minutos y la prueba se da por finalizada cuando se terminan las 48 tarjetas*.* Para la corrección se puntúa el número de categorías correctas, el número de errores perseverativos, de errores no perseverativos y de errores totales^31^.
*Memoria de trabajo*: se define como la habilidad cognitiva que nos permite mantener, manipular y usar información de manera temporal para realizar tareas complejas, como resolver problemas, planificar o tomar decisiones^32^. Para la evaluación de esta función, se seleccionaron las subpruebas dígitos directos, dígitos inversos y letras y números de la Escala de inteligencia de Wechsler para adultos-IV (WAIS-IV), siguiendo las normas propuestas por el manual^33^.
*Dígitos Directos (DD)*: la prueba consiste en que la persona evaluadora dice en voz alta secuencias de números que la persona debe repetir en el mismo orden. Con cada acierto, se incrementa la dificultad añadiendo un dígito. En caso de error, se proporcionan dos intentos por cada secuencia. El rango de puntuación es de 0 a 9^34^.
*- Dígitos Inversos (DI)*: la persona evaluadora dice en voz alta secuencias de números, pero esta vez, la persona debe repetir la secuencia en orden inverso, es decir, del último dígito al primero. En caso de error, se proporcionan dos intentos por cada secuencia. El rango de puntuación es de 0 a 8^34^.
*- Letter-Number Sequencing (LyN)*: en este último ejercicio las secuencias están formadas por números y por letras. El sujeto debe ordenarlas, reproduciendo de manera oral primero los números en orden creciente, y seguidamente las letras en orden alfabético. En caso de error, se proporcionan dos intentos por cada secuencia. El rango de puntuación es de 0 a 8^34^.
*Velocidad de procesamiento*: se define como la capacidad de detectar y responder de forma rápida y óptima a los cambios producidos en el entorno, como la presencia de un estímulo^35^. Para la evaluación de esta función, se selecciona la *parte A del Trail Making Test (TMTA),* en el que la persona debe unir con lápiz o bolígrafo números del 0 al 25 distribuidos aleatoriamente de en una hoja de papel. La puntuación es el tiempo de ejecución total en segundos^36^.
*Control inhibitorio*: se define como la capacidad de inhibir impulsos o conductas prepotentes para alcanzar objetivos específicos o seguir reglas establecidas^37^. Para la evaluación de este constructo se seleccionó la *parte B del Trail Making Test (TMTB),* que consiste en unir de forma alterna números y letras (los números de menor a mayor y las letras en orden alfabético), de tal forma que la secuencia sea 1, A, 2, B, 3, C... La puntuación es el tiempo total en segundos^36^.

### Análisis estadístico

Se calcularon las frecuencias y porcentajes de las variables categóricas tanto globales como desagregadas por sexo. Las variables categóricas se compararon entre grupos mediante la prueba Chi-cuadrado (*Χ*^2^) y el tamaño de efecto de las diferencias se valoró con V de Cramer (> 0,1 pequeño, > 0,3 moderado, > 0,5 grande)^38^. Las variables cuantitativas se describieron mediante media y desviación típica (DT), globalmente y desagregadas por sexo. Las diferencias por grupos fueron analizadas mediante la prueba estadística no paramétrica U de Mann-Whitney, junto con el tamaño del efecto r de Rosenthal (> 0,1 pequeño, > 0,3 moderado, > 0,5 grande)^39^.

Se valoró el efecto de distintas variables predictoras (primero las antropométricas, y en segundo lugar las psicológicas) sobre las puntuaciones obtenidas en las distintas variables cognitivas mediante regresión lineal múltiple por pasos hacia atrás, eliminando progresivamente las variables que no cumplían con el criterio de significación establecido en p < 0,05. El efecto ajustado se presentó con el coeficiente B y su intervalo de confianza (IC) al 95%. Se utilizó R^2^ como indicador del tamaño del efecto (> 0,02 pequeño, > 0,13 moderado, > 0,26 grande)^38^^,^^40^. Además, se realizaron correlaciones de Pearson para detectar posibles colinealidad (asociaciones lineales bivariadas entre pares de variables incluidas en los modelos).

Todos los análisis estadísticos se realizaron con el programa IBM SPSS Statistics for Windows v.21.0. (IBM Corp, Armonk, NY; 2012). Valores de p < 0,05 se consideraron significativos.

## RESULTADOS

La muestra estuvo compuesta por un total de 48 participantes españoles, con media de edad 47,58 años (DT=10,29), 25 mujeres (52,1%) y 23 varones (47,9%). La mayoría de la muestra son personas casadas (66,7%), con estudios secundarios (58,3%) y en situación de empleo (66,7%) (Tabla 1). No se encontraron diferencias estadísticamente significativas entre hombres y mujeres en relación a ninguna de estas variables.

**Tabla 1:** Datos descriptivos y frecuencias sociodemográficas totales y por sexo

	Total	Hombres	Mujeres	p	ES (V de Cramer)
	n=48	n=23 (47,9%)	n=25 (52,1%)		
	n (%)				
Edad (años)*	47,58 (10,38)	49,13 (10,266)	46,16 (10,503)	0,269	0,286^&^
*Estado civil*					
Solteras	10 (20,8)	4 (17,4)	6 (24)	0,305	0,194
Casadas	32 (66,7)	14 (60,9)	18 (72)		
Divorciadas	6 (12,5)	5 (21,7)	1 (4)		
*Nivel educativo (estudios)*					
Primarios	8 (16,7)	1 (4,3)	7 (28)	0,082	0,228
Secundarios	28 (58,3)	16 (69,6)	12 (48)		
Superiores	12 (25)	6 (26,1)	6 (24)		
*Situación laboral*					
Empleo	32 (66,7)	14 (60,9)	18 (72)	0,701	0,122
Desempleos	12 (25)	7 (30,4)	5 (20)		
Jubilación	4 (8,3)	2 (8,7)	2 (8)		

*: media (desviación típica); ES: *effect size* (tamaño de efecto); &: tamaño de efecto r de Rosenthal.

La Tabla 2 muestra los datos descriptivos cognitivos, antropométricos y psicológicos de la muestra total y desagregados por hombres y mujeres. Los resultados mostraron diferencias estadísticamente significativas por sexo en las variables antropométricas peso, IMC y masa grasa (kg y %) que fueron mayores en mujeres, con tamaños de efecto medianos.

En la Tabla 3 se muestran las correlaciones entre las variables antropométricas y el rendimiento en las pruebas ejecutivas. Se observaron diferentes correlaciones estadísticamente significativas: el porcentaje de MG se correlacionó de forma negativa con las puntuaciones en DD, DI y LyN, y de forma positiva con el tiempo empleado en completar el TMTB. El porcentaje de MM correlacionó positivamente con la puntuación en DD, DI y LyN, y negativamente con el tiempo para completar el TMTA y el TMTB.

**Tabla 2:** Datos descriptivos cognitivos, antropométricos y psicológicos globales y por sexo

	Total	Hombres	Mujeres	p	ES
	n=48	n=23 (47,9%)	n=25 (52,1%)		
	Media (DT)				
*Variables Cognitivas*					
*Test Modificado de Clasificación de Tarjetas de Wisconsin (M-WCST) (puntuación)*					
Categorías completas	4,60 (1,39)	4,78 (1,38)	4,44 (1,41)	0,369	0,13
*Errores*					
perseverativos	8,23 (5,30)	7,83 (5,33)	8,60 (5,37)	0,598	0,08
no perseverativos	6,54 (1,20)	6,39 (1,11)	6,68 (1,28)	0,352	0,13
totales	14,75 (5,45)	14,22 (5,49)	15,24 (5,47)	0,508	0,1
*Escala de inteligencia de Wechsler para adultos-IV (WAIS-IV) (puntuación)*					
Dígitos directos	5,33 (1,19)	5,39 (1,07)	5,28 (1,30)	0,554	0,09
Dígitos inversos	4,17 (1,15)	4,09 (0,99)	4,24 (1,30)	0,568	0,08
Letras y números	4,67 (1,15)	4,65 (0,88)	4,68 (1,37)	0,83	0,03
*Trail Making Test (TMT) (tiempo en segundos)*					
Parte A	31 (12,77)	32,78 (10,29)	29,36 (14,71)	0,102	0,24
Parte B	79,83 (38,22)	80,26 (31,92)	79,44 (43,88)	0,515	0,09
*Variables Antropométricas*					
Peso (kg)	115,70 (23,14)	108,40 (23,99)	122,41 (20,61)	0,035	0,3
IMC (kg/m^2^)	41,63 (8,13)	38,77 (6,48)	44,26 (8,71)	0,038	0,3
Grasa Visceral	17,42 (6,47)	17,13 (8,49)	17,68 (3,95)	0,242	0,17
Masa grasa (%)	45,071 (7,22)	42,67 (2,96)	47,28 (9,14)	0,032	0,31
Masa grasa (kg)	53,08 (16,89)	46,38 (11,18)	59,23 (19,02)	0,021	0,33
Masa muscular (%)	51,96 (7,05)	54,01 (3,87)	50,07 (8,71)	0,06	0,27
Masa muscular (kg)	59,81 (11,11)	59,16 (12,76)	60,40 (9,57)	0,556	0,08
*Variables Psicológicas (puntuación en Escala de Depresión, Ansiedad y Estrés, DASS21)*					
Total	16,31 (13,95)	14,70 (12,75)	17,80 (15,07)	0,522	0,09
Ansiedad	4,33 (4,49)	3,57 (3,38)	5,04 (5,28)	0,416	0,12
Estrés	6,48 (4,52)	6,04 (4,50)	6,88 (4,59)	0,475	0,1
Depresión	5,5 (5,82)	5,09 (5,64)	5,88 (6,07)	0,499	0,1

DT: desviación típica; ES*: effect size* (tamaño de efecto r de Rosenthal). En negrita, resultados estadísticamente significativos.

**Tabla 3:** Correlación de variables antropométricas y psicológicas con el rendimiento ejecutivo en personas con obesidad

	M-WCST				DD	DI	LyN	TMT	
	C	EP	ENP	ET				A	B
*Variables antropométricas*									
Peso (kg)	-0,048	0,023	0,103	0,045	-0,037	-0,086	0,023	-0,228	-0,131
IMC (kg/m^2^)	-0,181	0,17	0,021	0,17	-0,157	-0,181	-0,133	-0,03	0,195
Grasa visceral	-0,066	0,054	0,058	0,066	0,02	-0,078	0,022	-0,099	0,004
MG (%)	-0,192	0,195	-0,084	0,169	-0,324	-0,265	-0,273	0,214	0,322
MG (kg)	-0,11	0,1	0,019	0,1	-0,209	-0,181	-0,097	-0,068	0,072
MM (%)	0,24	-0,198	0,033	-0,183	0,363	0,284	0,299	-0,293	-0,344
MM (kg)	0,082	-0,123	0,174	-0,08	0,208	0,119	0,227	-0,353	-0,373
*Variables psicológicas (DASS-21)*									
Ansiedad	-0,335	0,251	0,202	0,291	-0,391	-0,355	-0,331	0,164	0,236
Estrés	-0,128	0,088	0,139	0,12	-0,322	-0,248	-0,242	0,096	0,19
Depresión	-0,271	0,153	0,167	0,19	-0,365	-0,339	-0,218	0,186	0,293

M-WCST: Test Modificado de Clasificación de Tarjetas de Wisconsin; C: categorías completas; EP: errores perseverativos; ENP: errores no perseverativos; ET: errores totales; DD: dígitos directos; DI: dígitos inversos; LyN: letras y números; TMT: Trail Making Test; A: parte A; B: parte B; IMC: índice de masa corporal; MG: masa grasa; MM: masa muscular; DASS-21: Escala de Depresión, Ansiedad y Estrés. En negrita, resultados estadísticamente significativos.

Los modelos de regresión múltiple realizados para cada una de las pruebas cognitivas utilizando como variables predictoras las variables antropométricas mostraron influencia significativa sobre las puntuaciones obtenidas en DD, LyN, y el tiempo empleado en completar las partes A y B del TMT (Tabla 4). El porcentaje de MM influye significativamente sobre la puntuación de DD. El modelo formado por las variables peso, MG (kg), MM (kg) y MM (%) influyó significativamente sobre la puntuación de LyN. La grasa visceral, MG (kg) y MM (%) influyeron significativamente sobre el tiempo de ejecución del TMTA. Y el IMC, la MG (kg) y la MM (%) influyeron significativamente sobre el tiempo de ejecución del TMTB. Los modelos de DD y LyN obtuvieron un tamaño del efecto mediano, mientras que los modelos del TMTA y TMTB obtuvieron un tamaño del efecto grande.

**Tabla 4:** Variables antropométricas como predictores de funciones ejecutivas (regresión lineal múltiple)

Variable criterio	B (IC95%)	p	ES (R^2^) / p del modelo
Variables predictoras			
*DD*			*0,132 / 0,011*
MM (%)	0,36 (0,01-0,10)	0,011	
*LyN*			*0,246 / 0,009*
Peso (kg)	-5,42 (-0,47 - -0,07)	0,009	
MG (kg)	4,45 (0,09-0,51)	0,005	
MM (kg)	2,63 (0,05-0,49)	0,016	
MM (%)	0,75 (-0,00-0,25)	0,061	
*TMT A*			*0,380 / <0,001*
Grasa visceral	0,44 (0,15 - 1,61)	0,018	
MG (kg)	-1,22 (-1,35 - -0,50)	<0,001	
MM (%)	-1,16 (-2,92 - -1,27)	<0,001	
*TMT B*			*0,380 / <0,001*
IMC (kg/m^2^)	1,10 (2,30-8,11)	0,001	
MG (kg)	-1,58 (-5,23 - -1,95)	<0,001	
MM (%)	-0,83 (-6,55 - -2,47)	<0,001	

B: coeficiente estandarizado; IC: intervalo de confianza; ES: tamaño de efecto; R^2^: coeficiente de determinación; DD: Dígitos Directos; LyN: Letras y Números; TMTA: *Trail Making Test* parte A; TMTB*: Trail Making Test* parte B, IMC: índice de masa corporal, MG: masa grasa; MM: masa muscular. En negrita, resultados estadísticamente significativos.

Los modelos de regresión múltiple realizados para cada una de las pruebas cognitivas utilizando como variables predictoras las variables psicológicas mostraron influencia significativa sobre las puntuaciones obtenidas en M-WCST-C, M-WCST-ET, DD, DI, LyN, y el tiempo empleado en completar el TMTB (Tabla 5). La ansiedad fue la única variable que influyó significativamente sobre las puntuaciones obtenidas en M-WCST-C, M-WCST-ET, DD, DI y LyN. Además de la depresión, la cual influyó de forma significativa con el TMTB. Todos los modelos obtuvieron tamaños del efecto medianos.

**Tabla 5:** Variables psicológicas como predictores de funciones ejecutivas (regresión lineal múltiple)

Variable criterio	B (IC95%)	p	ES (R^2^) / p del modelo
Variables predictoras			
*M-WCST-C*			*0,112 / 0,020*
Ansiedad	-0,33 (-0,19 - -0,01)	0,02	
*M-WCST-ET*			*0,085 / 0,045*
Ansiedad	0,29 (0,00-0,69)	0,045	
*DD*			*0,153 / 0,006*
Ansiedad	-0,39 (-0,17 - -0,03)	0,006	
*DI*			*0,107 / 0,013*
Ansiedad	-0,35 (-0,16 - -0,02)	0,013	
*LyN*			*0,109 / 0,022*
Ansiedad	-0,33 (-0,15 - -0,01)	0,022	
*TMTB*			*0,086 / 0,043*
Depresión	0,29 (0,06-3,78)	0,043	

B: coeficiente estandarizado; IC: intervalo de confianza; ES: tamaño de efecto; R^2^: coeficiente de determinación; M-WCST: Test Modificado de Clasificación de Tarjetas de Wisconsin; -C: categorías completas; -ET: errores totales; DD: Dígitos Directos; DI: Dígitos Inversos; LyN: Letras y Números; TMTB: *Trail Making Test* parte B. En negrita, resultados estadísticamente significativos.

## DISCUSIÓN

El objetivo de este estudio fue examinar la relación entre las variables antropométricas (peso, IMC, grasa visceral, grasa corporal y masa muscular) y el estado de salud psicológico (estrés, ansiedad y depresión) con el rendimiento ejecutivo (flexibilidad cognitiva, memoria de trabajo, velocidad de procesamiento y control inhibitorio) en personas con obesidad.

El presente artículo, a diferencia de estudios previos, ofrece un enfoque innovador e integral, al incorporar el análisis asociativo de variables antropométricas y psicológicas con el rendimiento de las funciones ejecutivas en personas con obesidad. Esta aproximación permite un análisis más detallado y preciso de cómo las características físicas y el estado de salud psicológico se relacionan con las funciones ejecutivas, proporcionando una comprensión más profunda de la interacción entre estos factores.

Los análisis realizados comparando las variables antropométricas, psicológicas y cognitivas por sexo, evidencian que hubo diferencias estadísticamente significativas en el peso, IMC y MG (kg y %) a favor de las mujeres. Esto concuerda con la literatura científica existente, donde se reporta que las mujeres presentan mayores niveles de grasa corporal en comparación con los hombres incluso cuando ambos presentan valores similares de peso e IMC. Esta diferencia responde a mecanismos biológicos, hormonales y evolutivos. Concretamente, los niveles más elevados de estrógenos en mujeres favorecen el almacenamiento de grasa subcutánea, especialmente en regiones glúteo-femorales, con una función adaptativa relacionada con la reproducción, el embarazo y la lactancia^41^.

En cuanto a los modelos de regresión, se reportan resultados interesantes en la relación entre las variables antropométricas y el rendimiento de las funciones ejecutivas. Variables como el peso, el IMC, la grasa visceral, la MM o la MG se han identificado como variables predictoras, las cuales influyen significativamente sobre el rendimiento de la memoria de trabajo (DD y LyN), la velocidad de procesamiento (TMTA) y el control inhibitorio (TMTB), vinculación reforzada por las asociaciones lineales significativas de los porcentajes de MG y de MM con estas funciones ejecutivas.

La literatura científica reciente proporciona evidencia que podría explicar este hecho, aludiendo a que los déficits cognitivos responden principalmente a los cambios sufridos en la morfología cerebral de las personas con obesidad^42^. A nivel neurofisiológico, el aumento del IMC y de la grasa corporal genera una activación del sistema inmunológico que provoca una inflamación crónica de bajo grado que desencadena la acumulación de proteína amiloide y citoquinas, y que se ha vinculado con el deterioro cognitivo de las funciones ejecutivas en estudios correlacionales^43^, longitudinales^44^, y experimentales^45^. Una de las zonas cerebrales más afectada por la acumulación de estas citoquinas tóxicas es la corteza prefrontal dorsolateral^46^, encargada de funciones como la memoria de trabajo y la flexibilidad cognitiva^47^^,^^48^.

Mediante estudios de neuroimagen se han detectado en el cerebro de personas con obesidad alteraciones estructurales y funcionales que afectan a regiones clave asociadas con las funciones ejecutivas. En términos funcionales, se ha observado una reducción en la conectividad en los circuitos frontoestriato-parietales, lo que podría limitar la integración y coordinación de información necesaria para procesos de control inhibitorio y toma de decisiones^49^. Desde un punto de vista estructural, estas alteraciones incluyen menor densidad de sustancia gris en subdivisiones de la corteza prefrontal, como la circunvolución frontal superior, el giro frontal inferior y la corteza orbitofrontal, regiones clave en procesos ejecutivos de planificación, flexibilidad cognitivo, control inhibitorio y resolución de problemas^50^^,^^51^. Además, en estudios con resonancia magnética funcional con realización de tareas que requieren función ejecutiva, se ha documentado la hipoactivación en la circunvolución frontal interior de la corteza prefrontal, lo que sugiere una disminución en la capacidad de estas áreas para responder de manera adecuada a las demandas cognitivas^52^.

Se ha de tener en cuenta también, la relación bidireccional entre los parámetros de la obesidad y las funciones ejecutivas, ya que otra posible explicación es la asociación entre la obesidad y la densidad de la materia blanca en el cerebro^53^. El IMC y porcentaje de MG se asocian con menor densidad en zonas del núcleo caudado, putamen y globo pálido, estructuras relacionadas con el control del comportamiento y el procesamiento de recompensas^54^. Un estudio reciente sobre conducta alimentaria informó que un déficit en la memoria de trabajo se asocia a la elección de alimentos más calóricos, especialmente *snacks* y grasas^26^, provocando un comportamiento menos flexible y más impulsivo^55^.

Nuestros resultados también han observado una relación inversamente proporcional entre la masa muscular y las funciones ejecutivas, señalando que a mayor MM (tanto % como kg), mejor rendimiento en tareas de flexibilidad cognitiva, memoria de trabajo, velocidad de procesamiento y control inhibitorio. La MM tiene un efecto directo sobre la condición física, mejorando atributos como la movilidad, la fuerza y la coordinación^56^, el bienestar psicológico^57^, la calidad de vida percibida^58^, y un menor riesgo de mortalidad^59^. La pérdida de MM, denominada sarcopenia, se ha relacionado con déficits cognitivos^60^, incluso con deterioro cognitivo leve^61^. Distintos estudios epidemiológicos sugieren que existen mecanismos fisiopatológicos relacionados entre la MM y los marcadores inflamatorios, los cuales subyacen el deterioro cognitivo. Por ejemplo, niveles más altos de interleucina-6 y proteína C reactiva se asociaron con la pérdida de músculo esquelético^62^ y fuerza muscular^63^. Además, un menor porcentaje de MM se vincula de forma significativa con alteraciones vasculares (menor velocidad de la onda del pulso braquial-tobillo), hiperintensidades de la materia blanca^64^, y atrofia cerebral cortical^65^. Los resultados de nuestro estudio respaldan la evidencia previa que relaciona un menor porcentaje de masa muscular con el deterioro cognitivo.

Los resultados también han aportan evidencia de la relación entre el estado de salud psicológico y el rendimiento en las funciones ejecutivas. La ansiedad, principalmente, y la depresión se han mostrado como variables predictoras de la flexibilidad cognitiva (M-WCST-C y M-WCST-ET), memoria de trabajo (DD, DI y LyN) y control inhibitorio (TMTB). Además, en las correlaciones se muestran asociaciones significativas inversamente proporcionales entre el rendimiento cognitivo y la ansiedad, el estrés y la depresión: a mayor sintomatología, peores resultados en las pruebas ejecutivas. Estos resultados concuerdan con la evidencia científica recogida hasta el momento, en la que múltiples metaanálisis demuestran como el estado depresivo empeora la memoria episódica^66^, la función ejecutiva^67^ y la atención^68^, mientras que el estrés se asocia con alteraciones atencionales, mnésicas y lingüísticas^69^.

La ansiedad es la variable psicológica que más relación mostró con el rendimiento ejecutivo tanto en los modelos de regresión como en las correlaciones. La literatura recoge evidencia sobre cómo esta asociación es bidireccional, ya que la obesidad puede aumentar la sintomatología ansiosa debido a factores metabólicos y sociales pero la ansiedad también puede conducir al aumento de peso a través del consumo emocional y alteraciones en el sistema de recompensa^70^. Con respecto al impacto en las funciones ejecutivas, existen mecanismos que pueden explicar cómo diversas alteraciones cerebrales provocadas por los procesos de ansiedad afectan a los procesos cognitivos. En primer lugar, la hiperactivación de la amígdala, característica en personas con ansiedad, interfiere con la comunicación de la corteza prefrontal, lo que dificulta la regulación de emociones y el control inhibitorio^71^. Este desequilibrio limita la capacidad de priorizar y procesar información relevante en situaciones de demanda cognitiva. En segundo lugar, en los procesos ansiosos existe una disfunción de la corteza prefrontal, particularmente en regiones dorsolaterales y ventromediales, las cuales comprometen el rendimiento de la memoria de trabajo, la planificación y la toma de decisiones^72^. En tercer lugar, la conectividad reducida entre la amígdala y regiones prefrontales intensifica las respuestas automáticas, lo que reduce la flexibilidad cognitiva necesaria para la adaptación a los cambios en el entorno^73^. Finalmente, la activación crónica del eje hipotalámico-hipofisario-adrenal (HHA) en personas con ansiedad eleva los niveles de cortisol y genera desequilibrios en neurotransmisores como GABA, serotonina y dopamina. Estos fenómenos neurotóxicos afectan a estructuras clave como el hipocampo y la corteza prefrontal, exacerbando los déficits en la memoria y aprendizaje lo que contribuye a un deterioro global en las funciones ejecutivas^74^.

A pesar de los hallazgos significativos de nuestro estudio, es importante reconocer varias limitaciones. En primer lugar, la naturaleza transversal del estudio impide establecer relaciones causales definitivas entre las variables antropométricas, el estado psicológico y el rendimiento de las funciones ejecutivas evaluadas. En segundo lugar, el tamaño muestral es pequeño, lo que hace que las pruebas estadísticas carezcan de la potencia suficiente para detectar otras posibles relaciones existentes. En tercer lugar, haber realizado el estudio con personas de dos centros de una misma ciudad puede limitar la generalización de los resultados. Por último, hay funciones ejecutivas que no se han evaluado de forma directa, como la planificación o la toma de decisiones. Para futuras investigaciones se recomienda utilizar diseños longitudinales, muestras más grandes y un protocolo de evaluación neuropsicológico más completo, con el objetivo de confirmar estos hallazgos y explorar los mecanismos subyacentes con mayor precisión.

En conclusión, los hallazgos del presente estudio subrayan la necesidad de considerar la composición corporal y el estado de salud psicológico como factores clave en la evaluación del rendimiento cognitivo en personas con obesidad. La influencia significativa del peso, el IMC, la grasa visceral y, especialmente de la MG y MM, sobre la memoria de trabajo, la velocidad de procesamiento y el control inhibitorio sugiere que los efectos de la obesidad van más allá de las características antropométricas, afectando también a la cognición. Asimismo, la ansiedad y la depresión mostraron asociaciones negativas con el rendimiento ejecutivo, los cual refuerza la importancia de adoptar enfoques integrales y multidisciplinarios en el abordaje clínico de esta población, que contemplen tanto intervenciones físicas como psicológicas para mejorar la calidad de vida.

## Data Availability

Los datos que respaldan los resultados de este estudio se encuentran disponibles, previa solicitud razonable a través de la autora de correspondencia.
